# Extrapulmonary tuberculosis presenting as hemorrhagic pleuro‐pericardial effusions with pericardial mass

**DOI:** 10.1002/ccr3.8619

**Published:** 2024-03-31

**Authors:** Mohammad S. Abdelghani, Ammar Chapra, Bara Al‐Qudah, Ahmed Bishawi, Ahmed Shebani, Ibrahim Obeidat, Mhd Baraa Habib

**Affiliations:** ^1^ Department of Cardiology Heart Hospital, Hamad Medical Corporation Doha Qatar; ^2^ Department of Internal Medicine Hamad Medical Corporation Doha Qatar; ^3^ Infectious Disease Division, Department of Internal Medicine Hamad Medical Corporation Doha Qatar

**Keywords:** extrapulmonary TB, pericardial effusion, pericardial masses, tuberculosis

## Abstract

**Key Clinical Message:**

Tuberculosis (TB) pericarditis, while uncommon, should be considered in patients with pericardial masses and effusion. Timely recognition and treatment with anti‐TB medications are crucial for a successful outcome.

**Abstract:**

TB pericarditis presenting as a pericardial mass is an unusual and rare manifestation of this disease. We report a 59‐year‐old South Asian male who presented with a 1‐week history of dyspnea and cough. He was found to have a hemorrhagic pericardial mass with a massive pericardial effusion. Pleural fluid analysis was positive for TB. The patient was successfully treated with anti‐TB medications. Although rare, tuberculous pericardial involvement should be suspected in patients presenting with symptoms of pericardial masses and effusion.

## 1 INTRODUCTION

Globally, tuberculosis (TB) ranks as the 13th leading cause of death and stands among the primary causes of mortality from a single infectious agent (excluding HIV/AIDS and COVID‐19). In 2022, an estimated 10.6 million people worldwide contracted TB.^1^ While the lungs constitute the primary site for Mycobacterium TB primary infection, about 20% of cases involve extrapulmonary TB, with lymphadenitis being the most prevalent site, followed by pleural TB.^2^ However, TB pericarditis only constitutes 1%–4% of all cases.^3^, ^4^

The route of entry occurs through inhaling aerosol droplets containing mycobacteria, leading to deposition in the lungs. This can result in one of four outcomes: immediate clearance of the organism, immediate active disease onset, latent infection, or reactivation of the disease, sometimes years after latency.^5^

Pericardial infection with Mycobacterium TB can stem from lung, tracheobronchial tree, adjacent lymph nodes, spine, sternum extension, or a hematogenous route through miliary spread. Tuberculous pericarditis often signifies reactivation disease, and the primary infection site might not be evident.^6^

Instances of pericardial mass combined with hemorrhagic effusions as a manifestation of TB pericarditis are infrequently documented in medical literature. This case highlights an unusual presentation of extrapulmonary TB, ultimately diagnosed as pleural‐pericardial TB.

## 2 CASE REPORT

### 2.1 Case history and examination

A 59‐year‐old South‐Asian male, with no history of chronic illnesses, presented to the Emergency Department with complaints of severe shortness of breath, dry cough, chest discomfort, and palpitations lasting for 1 week, worsening in the last 2 days before presentation. There was no fever, chills, rhinorrhea, or contact with sick individuals. Further inquiry revealed decreased appetite, subjective weight loss, and no night sweats. Although he had no personal history of TB, his father had been treated for pulmonary TB three decades earlier. He was not known to be on any chronic medications, did not possess any known allergies, and had never smoked cigarettes nor consumed alcohol.

Upon initial assessment, he exhibited mild respiratory distress with a respiratory rate of 35 breaths per minute and struggled to complete full sentences. Vital signs indicated tachycardia (153 beats per minute), blood pressure of 153/103 mmHg, and oxygen saturation of 97% without any oxygen support. Bilateral basal inspiratory crackles, muffled heart sounds, and elevated jugular venous pressure were observed during chest and cardiovascular examinations, but no pedal edema was noted.

### 2.2 Investigations and treatment

An ECG displayed sinus tachycardia without ST‐T wave changes. Complete blood count on admission showed no leukocytosis (8.1 × 10^3^/μL), microcytic hypochromic anemia with hemoglobin of 10.8 g/dL, and platelets count 417 × 10^3^ μL. Liver functions test showed a normal serum bilirubin of 11 μmol/L, total protein 67 g/L, low serum albumin 22 g/L, and derangements in alkaline phosphatase 138 U/L, aspartate aminotransferase 67 U/L, and alanine aminotransferase 70 U/L. Renal function testing revealed normal urea (3.5 mmol/L) and normal creatinine (87 μmol/L). Also, there was mild hyponatremia (129 mmol/L) with mild hypokalemia (3.2 mmol/L). Serology of HCV antibody, HBsAg, and HIV 1 and 2 antibodies was negative.

**FIGURE 1:**
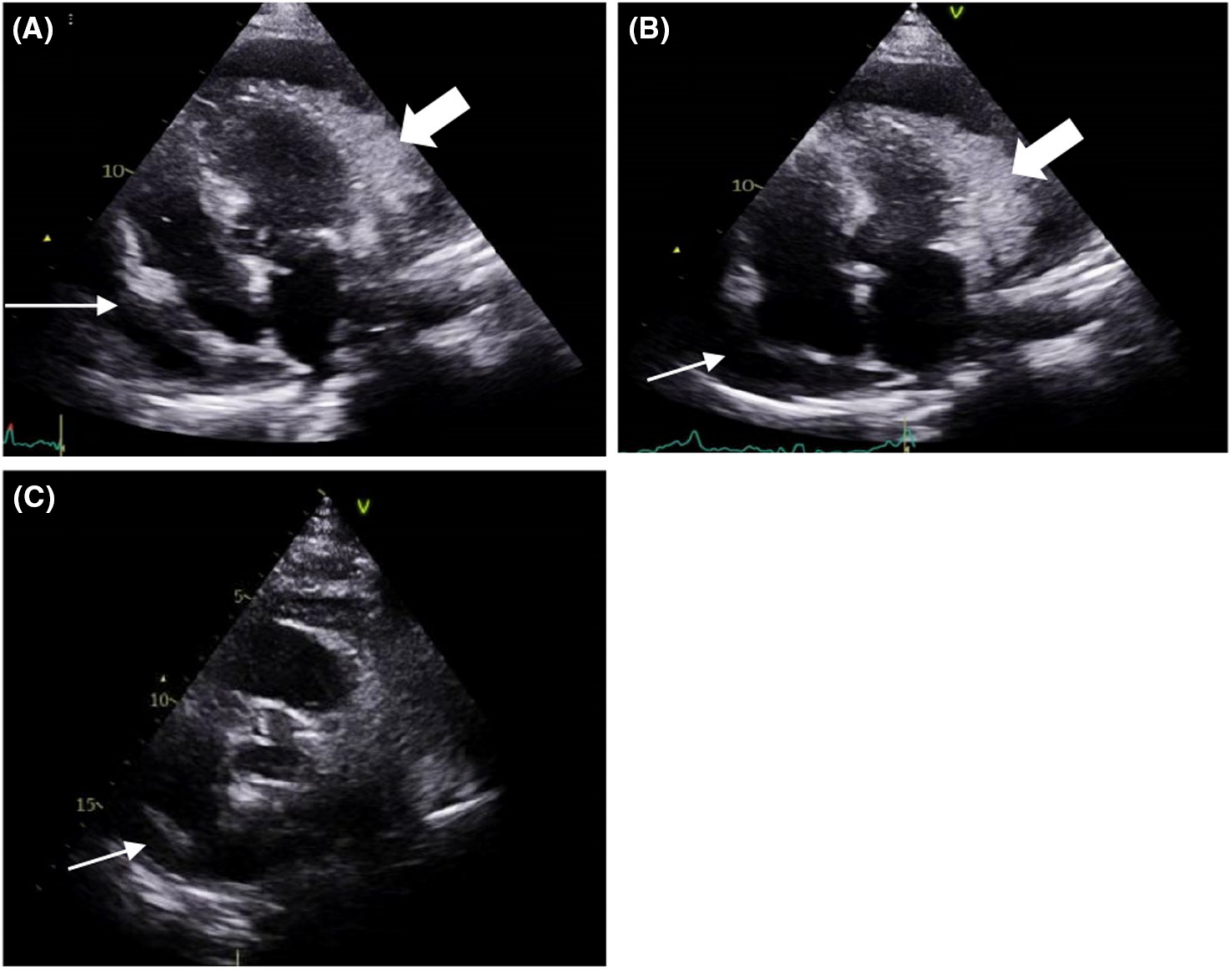
(A and B) Echocardiography upon presentation: showing a large pericardial effusion possibly fibrinous (thin white arrow) with respiratory variation less than 25%. It also revealed an echo‐dense structure seen attached to the visceral pericardium (thick white arrow). (C) Echocardiography post pericardiocentesis and after being started on antituberculous therapy: showing minimal pericardial effusion (thin white arrows).

Initially diagnosed with pulmonary edema, he received intravenous furosemide and nitrate infusions without immediate improvement. Subsequent chest radiography showed cardiomegaly with bilateral pleural effusions and blurred heart borders. Initial focused echocardiography was performed which confirmed a substantial pericardial effusion, along with an echo‐dense structure attached to the visceral pericardium, predominantly in the lateral and anterior parts of the left ventricle (LV) with fibrin shreds (Figure 1A,B).

After transfer to the cardiac intensive care unit, pericardiocentesis through a parasternal window drained 500 mL of sanguineous effusion. Analysis indicated hemorrhagic effusion with lymphocytic predominance, yet bacterial and TB tests, including acid‐fast bacilli (AFB) smear, TB PCR, and TB culture, returned negative results (Table 1). The possibility of underlying malignancy persisted. Follow‐up complete echocardiographic study was later performed which showed a normal global systolic LV function with an ejection fraction (EF) of 54% with a normal right ventricular function and no evidence of valvular pathology. Subtle wall motion abnormalities could not be ruled out due to poor image quality. There was residual minimal pericardial effusion seen. Subsequent chest and abdomen computed tomography (CT) scan revealed bilateral pleural and pericardial effusion without detectable masses or suspicious lesions. Further testing, including sputum AFB smears, TB PCR, and TB culture, which also yielded negative results. Serum QuantiFERON‐TB Gold Plus (QFT‐Plus) was reported as “indeterminate.” No additional screening tests for TB were performed.

**TABLE 1:** Pericardial and pleural fluid analysis.

Test	Pericardial fluid analysis	Pleural fluid analysis
Color	Red	Red
Appearance	Bloody	Bloody
WBC	1000/μL	1000/μL
RBC	247,125/μL	103,750/μL
Neutrophils	22.0%	20.0%
Lymphocytes	64.0%	65.0%
Monocytes	12.0%	12.0%
Mesothelial cells	1.0%	2.0%
Others	Cytology: Lymphocytic effusion No malignant cells	Glucose 5.5 mmol/L LDH 514.0 U/L
Fluid culture	No growth	Lab assay unavailable

Abbreviations: RBC, red blood cells; WBC, white blood cell count.

Given the need for further evaluation, pleural fluid analysis via thoracentesis (Table 1) demonstrated exudative hemorrhagic and predominantly lymphocytic effusion. Subsequent TB workup from the pleural effusion tested positive for Mycobacterium TB PCR through GeneXpert MTB/RIF, although subsequent TB culture did not yield viable results. With the diagnosis of pleural and presumptive pericardial TB, a four‐drug anti‐TB regimen, including rifampicin, isoniazid, pyrazinamide, and ethambutol, alongside pyridoxine, was initiated.

### 2.3 Outcome and follow‐up

The patient tolerated the treatment well with no reported adverse reactions. His condition improved significantly after pericardiocentesis and the initiation of anti‐TB medications. He was discharged in favorable clinical condition for outpatient follow‐up. Upon a 3‐week follow‐up, the patient reported weight gain and was asymptomatic. Echocardiography revealed minimal residual pericardial effusion (Figure 1C).

## 3 DISCUSSION

Despite the classically reported symptoms of TB infection being constitutional, such as fever, night sweats, and weight loss, along with symptoms related to the affected organs, there could be significant phenotypic heterogeneity. In our case, it manifested as a hemorrhagic pericardial effusion with a pericardial mass resembling malignancy.

Pericardial masses caused by TB are exceedingly rare, with only a few cases reported in medical literature so far.^4^, ^7^, ^8^, ^9^, ^10^, ^11^, ^12^ When discovered, pericardial masses are mostly attributed to malignancies, with metastatic pericardial involvement encountered more frequently than primary tumors, often resulting in a poor prognosis.^13^ However, in TB pericarditis, the pathophysiology of these masses remains poorly understood, with suggestions that they result from a conglomerate of red blood cells and protein materials within the pericardial fluid.^7^

TB pericarditis may present with congestive heart failure symptoms or, in late‐stage patients, with constrictive pericarditis symptoms. The four recognized stages of TB pericarditis are (1) fibrinous exudation with polymorphonuclear leukocytosis, abundant mycobacteria, and early granuloma formation with loose organization of macrophages and T cells (similar to the mass seen in our patient in Figure 1A,B). (2) Serosanguineous effusion with lymphocytic exudate and high protein concentration, with low concentrations of tubercle bacilli. (3) Effusion absorption with granulomatous caseation and pericardial thickening, followed by fibrosis. (4) Constrictive scarring; fibrosing visceral and parietal pericardium contracts on cardiac chambers, potentially leading to calcification and constrictive pericarditis, impeding diastolic filling.^8^

In this case, our patient hailed from an endemic TB area, and he exhibited symptomatic pericardial effusion with echocardiographic findings suggestive of a pericardial mass. There was no obvious explanation as to why this particular patient developed a pericardial mass in response to tuberculous pericarditis. The hypothesis generated from similar cases^8^ seems to be that in early stages of tuberculous pericarditis in young healthy adults such as our patient, a stronger immune reaction is likely mounted, leading to an early formation of a granuloma by recruiting large number of neutrophils, T cells, and macrophages which may mimic a mass seen in our patient. This hypothesis is supported by the fact that immune deficient patients such as those with HIV, granuloma formation with tuberculous pericarditis is much less than in immune‐competent individuals.^8^

Pericardial fluid analysis revealed hemorrhagic effusion with negative TB workup, prompting further investigation to rule out neoplasms. While a CT scan did not reveal chest or abdominal masses, it uncovered bilateral pleural effusions. Diagnostic pleurocentesis confirmed TB PCR positivity. TB emerged as the likely diagnosis due to the clinical presentation, positive PCR result, and improvement following initiation of antituberculous medications, making alternative diagnoses less likely and deferring the need for pericardial biopsy.

For diagnosis of any TB from any sample be it sputum, pleural fluid, or pericardial fluid, available modalities commonly utilized include acid‐fast bacilli microscopy, GeneXpert MTB/RIF Assay, and culture, with each of these offering variable sensitivity for detection. For pleural TB, pleural fluid adenosine deaminase and pleural biopsy have been reported to have the highest sensitivity (100% and 94.7% respectively) whereas pleural fluid culture and Xpert assay had low sensitivities being 45% and 25%, respectively.^14^ In our case, the GeneXpert Assay despite its low reported sensitivity was positive; however, it highlights the low yield of the commonly utilized tests which makes extrapulmonary TB a diagnostic challenge. Similarly for pericardial TB, the highest yield appears to be from pericardial biopsy (10%–64%) and TB PCR of pericardial tissue (80%) which are limited by their invasive nature, whereas culturing TB from pericardial fluid has a yield of (53%–75%).^15^

This case underscores the added value of analyzing pleural fluid, especially in cases with concurrent pleural effusion and pericardial mass. It also underscores echocardiography's utility as a precise diagnostic tool for pericardial masses, providing crucial data for decision‐making and effective treatment strategies. Regular follow‐up echocardiography is essential to monitor pericardial effusion progression or the development of constrictive pericarditis, potentially necessitating further drainage or surgical intervention.

## 4 CONCLUSION

Patients presenting with symptoms of pericardial effusion without a clear cause, especially those from regions where TB is prevalent, should be evaluated for possible tuberculous pericardial involvement. TB‐associated pericardial masses are exceedingly rare, underscoring the importance of maintaining a high level of suspicion to ensure accurate diagnosis, prompt initiation of anti‐TB treatment, and the prevention of repeated invasive procedures, such as pericardial biopsies.

## AUTHOR CONTRIBUTIONS

**Mohammad S. Abdelghani:** Conceptualization; writing – original draft; writing – review and editing. **Ammar Chapra:** Writing – original draft; writing – review and editing. **Bara Al‐Qudah:** Writing – original draft. **Ahmed Bishawi:** Writing – review and editing. **Ahmed Shebani:** Writing – review and editing. **Ibrahim Obeidat:** Writing – review and editing. **Mhd Baraa Habib:** Writing – review and editing.

## FUNDING INFORMATION

This research did not receive any specific grant from funding agencies in the public, commercial, or not‐for‐profit sectors.

## CONFLICT OF INTEREST STATEMENT

The authors report no conflict of interest.

## ETHICS STATEMENT

Ethical Approval was obtained by Medical Research Center (MRC) under ID MRC‐04‐23‐188.

## CONSENT

Written informed consent was obtained from the patient for publication of this case report and any accompanying images. A copy of the written consent is available for review by the Editor‐in‐Chief of this journal.

## Data Availability

Data can be obtained from the corresponding author upon request.
